# Clinical Characteristics and Management of Hypercalcemic Crisis in 155 Patients: A Single Center Retrospective Study

**DOI:** 10.1155/2024/4689745

**Published:** 2024-08-30

**Authors:** Yuqing Qu, Yang Liu, Xianling Wang, Qinghua Guo, Jin Du, Yu Pei, Jianming Ba, Weijun Gu, Jingtao Dou, Zhaohui Lv, Yiming Mu

**Affiliations:** ^1^ Department of Endocrinology The First Medical Center of Chinese PLA General Hospital, Beijing 100853, China; ^2^ Department of Endocrinology Yantai Yuhuangding Hospital, Yantai 264000, Shandong, China; ^3^ Department of Endocrinology Shexian Hospital, Handan 056400, Hebei, China

## Abstract

**Objective:**

This study aimed to analyse the etiology and clinical characteristics of hypercalcemic crisis in a large cohort of Chinese patients and summarised our clinical experience in the management of this serious endocrinological emergency.

**Methods:**

This was a retrospective analysis of a cohort of patients with hypercalcemic crisis hospitalized in the First Medical Center of Chinese PLA General Hospital between January 2009 and March 2024. The general data, clinical manifestations, etiology, photographic examination, emergency treatment, etiological treatment, and prognosis were analysed.

**Results:**

A total of 155 patients with hypercalcemic crisis (91 males and 64 females) with a mean age of 54.60 ± 16.99 years old were enrolled. The most frequent disease-causing hypercalcemic crisis was hyperparathyroidism (41.94%), followed by solid malignancy (41.29%) and multiple myeloma (9.03%), et al. Patients mainly presented with symptoms of the digestive system (78.10%), nervous system (63.30%), skeletal system (59.60%), urinary system (59.50%), and cardiovascular system (34.90%). These 155 patients with hypercalcemic crisis got effective therapies that included simultaneous administration of intravenous injection (IV) isotonic saline, subcutaneous calcitonin, bisphosphonate, or hemodialysis in serious cases. After emergency treatment, all the symptoms in the patients were relieved obviously. The cure rate of hypercalcemic with etiological treatments was 84.50% (131/155).

**Conclusion:**

Hypercalcemic crisis is a serious endocrinological emergency with a variety of etiologies and a high risk of mortality. A prompt diagnosis and the implementation of a comprehensive and effective treatment can efficiently alleviate this endocrinological emergency. Etiological treatment targeting different causes can improve prognosis significantly.

## 1. Introduction

Hypercalcemic is one of the most prevalent endocrine disorders with the total serum calcium (SCa) level above the upper limit of the normal reference range (usually above 10.5 mg/dl). The severity of hypercalcemic can be classified as mild (SCa <12 mg/dL or 3 mmol/L), moderate (SCa 12−14 mg/dL that is 3–3.5 mmol/L), and severe (SCa >14 mg/dL that is 3.5 mmol/L). The total SCa level above 14 mg/dL accompanied by a series of serious clinical signs is called a hypercalcemic crisis. Due to the wide spectrum of the emergency and the lack of specific constitutional symptoms, the association between hypercalcemic and its clinical manifestations has often been covered up by other primary diseases and ignored. It easily leads to misdiagnosis and delayed treatment. Therefore, emergency treatments are often required before the cause is discovered. Identification of the etiology of hypercalcemic and effective targeted therapy are essential to improve the prognosis [1, 2]. Till now, there have been few reports on the diagnosis and management of hypercalcemic crisis in a large sample of the Chinese population. In this report, we analysed the clinical data of 155 patients with hypercalcemic crisis in our hospital and summarized the characteristics, spectrum, and our clinical experience in the diagnosis and therapy of this serious endocrinological emergency.

## 2. Materials and Methods

### 2.1. Patients

One hundred and fifty-five patients with a confirmed diagnosis of hypercalcemic crisis between January 2009 and March 2024 were collected from the medical database of the First Medical Center of Chinese PLA General Hospital. Patients with the level of total SCa corrected for albumin above 14 mg/dL and accompanied by different clinical manifestations were involved.

### 2.2. Methods

All clinical information was retrospectively analysed as follows: (1) general information, such as age, gender, and presence of symptoms; (2) laboratory evaluation, including total SCa, serum phosphorus, alkaline phosphatase (ALP), serum 25-hydroxyvitamin D, parathyroid hormone (PTH); (3) tumour localization determined by various methodologies, including ultrasound, parathyroid computed tomography (CT), parathyroid magnetic resonance imaging (MRI), ^18^fluorine-labelled deoxyglucose positron emission computed tomography (^18^F-FDG PET-CT), parathyroid 99mTc methoxy isobutyl isonitrile (99mTc MIBI), digital X-ray bone imaging, dual-energy X-ray bone mineral density, and radionuclide bone imaging; (4) emergency therapy and etiological therapy (surgery, radiofrequency ablation, chemotherapy, radiotherapy, etc.); (5) pathological and immunohistochemical assessment of tumours; and (6) follow-up.

### 2.3. Statistical Analysis

Statistical analyses were performed with IBM SPSS Statistics version 22.0. Continuous data were presented as mean ± standard deviation. Quantitative data with nonnormal distribution were presented as median (*r*) or interquartile range (P25 and P75).

## 3. Results

### 3.1. General Information

A total of 155 patients (91 males and 64 females) were diagnosed with hypercalcemic crisis. The mean age was 54.60 ± 16.99 years old. Patients presented with digestive system symptoms (78.10%, mainly manifested as nausea, vomiting, and anorexia), nervous system symptoms (63.30%, mainly manifested as dizziness, fatigue, and slow response), skeletal system symptoms (59.60%, mainly manifested as bone pain, osteoporosis, and fractures), urinary system symptoms (59.50%, manifested as frequent urination, urgency, hematuria, and renal insufficiency), and cardiovascular system symptoms (34.90%, mainly manifested as palpitations, chest tightness, and chest pain).

### 3.2. Etiologies

The etiology of 155 patients indicated that hyperparathyroidism was the most common cause of hypercalcemic crisis (41.94%, 65/155), followed by solid malignancy (41.29%, 64/155), multiple myeloma (9.03%, 14/155), hypervitaminosis (1.29%, 2/155), familial hypocalciuric hypercalcemic (0.65%, 1/155), and unknown etiology (5.81%, 9/155).

#### 3.2.1. Hyperparathyroidism

The components of hyperparathyroidism were primary hyperparathyroidism (72.31%, 47/65) and tertiary hyperparathyroidism (27.69%, 18/65). The various systematic clinical manifestations of hypercalcemic crisis caused by primary hyperparathyroidism included the skeletal system, gastrointestinal system, urinary system, nervous system, and cardiovascular system. All patients with tertiary hyperparathyroidism had chronic renal failure with an average duration of 3.9 ± 0.2 years (Table 1).

Multiple noninvasive imaging and invasive imaging were performed to localise the tumour. The performance of ultrasound examination, parathyroid 99mTc methoxy isobutyl isonitrile (99mTc MIBI), CT scan, and MRI scan was 87.69% (57/65), 56.92% (37/65), and 12.31% (8/65), respectively. The positive detection rates were 68.42% (39/57) with ultrasound, 85.45% (47/55) with parathyroid 99mTc MIBI, 81.08% (30/37) with parathyroid CT, and 75.00% (6/8) with parathyroid MRI, respectively. Various examinations were carried out at different times in clinical practice, and patients underwent different examinations due to various reasons, and there were also differences in the examination rate and positive rate.

#### 3.2.2. Solid Malignancy

There were 64 patients with solid malignancies. In 75.00% (48/64) of the patients, the secondary hypercalcemic crisis was due to a long history of primary diseases. Oesophageal cancer, lung cancer, and leukemia accounted for 20.83% (10/48), 18.75% (9/48), and 12.50% (6/48), respectively. In addition to the primary tumour disease, clinical manifestations of other systems were also combined, with the digestive system being the most common, followed by the nervous system, cardiovascular system, and urinary system. Hypercalcemic crisis was the initial manifestation in 25.00% (16/64) of patients with solid malignancies, of which lung cancer accounted for 37.50% (6/16) and leukemia for 25.00% (4/16) (Table 1).

For tumour localization and bone metastasis identification, a variety of examinations were performed, including ^18^fluorine-labelled deoxyglucose positron emission tomography-computed tomography (^18^F-FDG PET-CT) and radionuclide bone imaging. The examination rates of 18F-FDG PET-CT and radionuclide bone imaging were 42.19% (27/64) and 23.44% (15/64), with positive rates of 100% (27/27) and 60% (9/15), respectively.

#### 3.2.3. Multiple Myeloma

Fourteen patients were diagnosed with multiple myeloma. Patients most commonly presented with skeletal system symptoms (78.57%, 11/14), digestive system symptoms and urinary system symptoms (71.42%, 10/14), nervous system symptoms (42.86%, 6/14), and cardiovascular system symptoms (35.71%, 5/14) (Table 1).

### 3.3. Diagnosis

A total of 3.87% (6/155) of patients experienced misdiagnosis or missed diagnosis. One case had acute abdominal symptoms, leading to a misdiagnosis of obstructive jaundice. Other 1.94% (3/155) of the patients had been initially revisited for malignancy (one case of liver metastasis from hepatocellular carcinoma and two cases of multiple myeloma). However, none of them presented with hypercalcemic symptoms known at the time of the early revisit. Upon admission, patients were referred to various departments, including endocrinology department (23.87%, 37/155), hematology department (20.00%, 31/155), hepatobiliary surgery department (17.42%, 27/155), oncology department (13.55%, 21/155), and thoracic department (12.26%, 19/155), et al.

With laboratory examinations (SCa, blood phosphorus, alkaline phosphatase, PTH, 25-OH vitamin D3, and other biochemical tests) and location (parathyroid ultrasound, parathyroid CT, parathyroid MRI, 18F-FDG PET-CT, parathyroid 99mTc MIBI, bone digital X-ray imaging, dual-energy X-ray bone mineral density, radionuclide bone imaging, etc.), the rapid diagnosis of hypercalcemic crisis (diagnosis days: 0.9 ± 0.1 days) was confirmed after admitting in our hospital, and the cause of this serious disorder was identified in 89.68% (139/155) of patients (interval time: 7.0 ± 0.3 days). Later, further targeted treatments were then performed.

### 3.4. Treatment

#### 3.4.1. Emergency Treatment

Among the 155 patients, emergency calcium-lowering treatments such as water intake (4000 ml of water per day) or hydration therapy (intravenous infusion of 4000 ml of normal saline) were performed in 78.80% (122/155) of patients, diuretic therapy in 56.70%, (88/155) of patients, calcitonin therapy in 45.10% (70/155) of patients, bisphosphonate therapy in 69.00% (107/155) of patients, glucocorticoids in 34.10% (53/155) of patients, and hemodialysis in 7.70% (12/155) of patients. After emergency treatment, the mean corrected SCa level in these patients decreased from 15.64 ± 1.96 mg/dL to 10.68 ± 2.08 mg/dL (normal range: 8.36–10.16 mg/dL). The treatment effectively reduced the SCa levels in all 155 patients with various causes of hypercalcemic crisis. The symptoms were all relieved significantly. The average duration of emergency treatment was 3.3 ± 1.2 days. No patient died from hypercalcemic crisis.

#### 3.4.2. Etiological Treatment and Outcome

65.95% (31/47) of patients with primary hyperparathyroidism underwent surgery, and 96.78% (30/31) of patients underwent postoperative pathology. The diagnosis of parathyroid adenoma was confirmed in 96.67% (29/30) of patients, and serum PTH (interquartile range) decreased from 721.70 (318.45, 1351.50) pg/ml to 15.22 (10.23, 37.28) pg/ml (normal range: 15–65 pg/ml), meanwhile the corrected SCa levels decreased from 15.40 ± 1.64 mg/dL to 11.16 ± 1.36 mg/dL. Clinical symptoms and signs were significantly alleviated. As a result, the surgical cure rate was 100% (31/31).

A total of 33.33% (6/18) of patients with triphasic hyperparathyroidism underwent surgery, and postoperative pathology (5/5) confirmed the diagnosis of parathyroid adenoma-like hyperplasia. The mean preoperative serum PTH level was 434.35 (262.32, 833.37) pg/mL, which decreased to 15.81 (13.11, 103.41) pg/mL after surgery, and the mean corrected postoperative SCa level decreased from 14.44 ± 2.44 mg/dL to 10.44 ± 0.88 mg/dL. Patients who underwent surgery showed significant improvement in clinical symptoms and signs. Forty-eight patients with a history of a primary solid malignancy and secondary hypercalcemic crisis underwent targeted treatment of the primary diseases. The response rate was 60.42% (29/48) in these patients treated with chemotherapy (62.50%, 30/48) and radiotherapy (6.25%, 3/48). In 16 patients with a primary malignancy, the response rate was 87.50% (14/16) after targeted treatment of the primary diseases. Among the 71.43% (10/14) of patients with multiple myeloma who were treated with chemotherapy, the response rate was 78.57% (11/14). With effective targeted therapy of the primary disease, SCa levels were significantly reduced and the associated symptoms were significantly alleviated in these patients.

## 4. Discussion

Hypercalcemic crisis is a serious life-threatening metabolic disorder, and the most common causes are primary hyperparathyroidism, solid malignancies, and multiple myeloma, accounting for over 90% of all precipitating factors. Other causes of hypercalcemic crisis are relatively rare [3, 4]. In a large sample of Chinese patients, our study identified that the most common conditions were hyperparathyroidism (41.94%), solid malignancies (41.29%), and multiple myeloma (9.03%). In hyperparathyroidism, PTH can activate the osteoclasts, leading to the release of large amounts of bone calcium into the bloodstream, promoting kidney reabsorption of calcium and the synthesis of more vitamin D, and causing the hypercalcemic crisis [5]. In patients with solid malignancies, in addition to osteolytic hypercalcemic caused by bone metastases, systemic fluid factors secreted by tumour cells, such as PTH-related protein (PTHrP), are important mechanisms. Such fluid factors can activate PTH receptors, leading to a hypercalcemic crisis.

The clinical manifestations of hypercalcemic crisis are highly variable and easily confused with other diseases, hence early recognition of the symptoms and signs of hypercalcemic crisis is critical. The onset of clinical symptoms and signs associated with hypercalcemic depends on factors such as the rate of calcium elevation, the degree of elevation, and the patient's tolerance to hypercalcemic [6]. The normal functioning of the nervous system requires an adequate SCa concentration. Therefore, high SCa can lead to cognitive dysfunction, ataxia, and even coma. The cardiovascular system exhibits hypertension, bradycardia, arrhythmias, and QT interval shortening. The gastrointestinal tract manifests as anorexia, nausea, vomiting, and constipation. The urinary system manifestations include polyuria, kidney stones, renal calcifications, decreased glomerular filtration rate, and hyperchloremic acidosis [7, 8]. In this study, the proportion of patients with cardiovascular manifestations was relatively low, indicating that cardiovascular complications may not be common in hypercalcemic crisis [9]. It has been reported in the literature that hypercalcemic crisis can usually be misdiagnosed as chronic gastritis or urinary tract stones [6]. The complex and variable clinical manifestations can easily lead to misdiagnosis. Therefore, specialists should be alert to the occurrence of a hypercalcemic crisis when analysing similar clinical manifestations. In addition, it is necessary to promptly monitor SCa levels in patients with malignant tumours to avoid misdiagnosis of hypercalcemic.

Primary hyperparathyroidism can be diagnosed on the basis of clinical presentations, bone lesions, renal lithiasis, and high SCa levels with concomitant high levels of PTH. Moreover, high alkaline phosphatase levels and urinary calcium excretion, low serum phosphorus, and specific X-ray image changes support such a diagnosis. Triple hyperparathyroidism occurs as a result of long-term secondary hyperparathyroidism [10]. Prolonged and intense stimulation of the parathyroid tissue leads to the development of autonomous hyperplasia or adenoma, resulting in SCa levels above normal and often requiring surgical intervention. With regard to the location of parathyroid adenomas, this study showed that 99mTc MIBI had a higher sensitivity compared to ultrasound and CT scans. Combining these two techniques could significantly improve the detection rate of tumours, particularly for ectopic and small adenomas [11].

Multiple myeloma is classified into monoclonal immunoglobulin G (IgG) excess syndrome of unknown significance, smoldering myeloma and active multiple myeloma. It is characterised by a tumour-like proliferation of plasma cells in the bone marrow, which stimulate osteoclasts and destroy bone tissues, leading to hypercalcemic, bone pain, and pathological fractures [12]. Fourteen patients were diagnosed with multiple myeloma after immunological tests and histological biopsies in different departments in this study.

In the treatment of hypercalcemic crisis, rapid reduction of calcium is of paramount importance, and etiological treatments play a key role. The emergency reduction of calcium involves various aspects, such as fluid infusion, promoting renal calcium excretion, preventing intestinal calcium absorption, reducing bone absorption, and so on. Fluid hydration is the initial therapy. Usually, 3 to 4 litres of normal saline should be infused within the first 24 hours. Blood electrolytes and electrocardiograms should be monitored frequently during fluid hydration to detect electrolyte disturbances. When blood volume is normalized, loop diuretics can be administered, while thiazide-type diuretics should be avoided.

Calcitonin inhibits bone resorption and increases urinary calcium excretion. It is effective in rapidly reducing calcium in patients with hypercalcemic crisis. Calcitonin 100–400 U injected once every 6 hours via either intravenously or subcutaneously is commonly administrated in 2-3 doses per day, but it often shows “escape” phenomena within a few hours or days and reduces its efficacy.

Intravenous bisphosphonates are potent inhibitors of osteoclast-mediated bone resorption and provide longer-term control of hypercalcemic than saline. Zoledronic acid, one of the most widely used and effective bisphosphonates, is highly recommended. Before zoledronic acid intravenous injection, it is important to ensure adequate hydration and pretreatment of drug heat. The effects of fluid resuscitation, diuretics, calcitonin, and dialysis treatment are rapid and short-lived. On the other hand, the effects of bisphosphonates and glucocorticoids are slow and take longer to reduce calcium [13]. Denosumab is a human monoclonal antibody targeting nuclear factor-*κ*B ligand-receptor activator. It acts as an antiresorption agent by reducing osteoclastogenesis and is a novel therapeutic approach for osteoporotic fractures and skeletal-related events in patients with high bone turnover [14]. Denosumab has been proven to reduce SCa levels. In this study, a total of 155 patients were treated with effective calcium-lowering therapies, and the cure rate was 100% (155/155). Hence, this may provide a favourable condition for subsequent targeted treatments.

Once the hypercalcemic crisis has been relieved with effective emergency therapies, etiological treatment can achieve a high cure rate of hypercalcemic and get a better prognosis [15]. The most effective treatment for patients with primary hyperparathyroidism is surgery. After the removal of the parathyroid tumour, hypercalcemic can be cured. For solid malignancies, various factors are evaluated and multiple treatment modalities including surgery, radiotherapy, chemotherapy, targeted therapy, and immunotherapy should be performed. For other causes of hypercalcemic, medication is usually the primary treatment, as reported in previous studies [1, 16]. In this study, all 37 patients with primary hyperparathyroidism underwent surgery and achieved a complete cure with normal serum PTH and SCa. Following targeted treatment in patients with solid malignancies, the level of SCa also decreased significantly. Forty-four patients with solid malignancies got better responses. The chemotherapy rate, improvement rate, and response rate in multiple myeloma patients were also significantly increased (Table 2).

In conclusion, our study retrospectively analysed a large sample of cases of hypercalcemic crisis. The disease spectrum of hypercalcemic crisis is various. The top three etiologies of the emergency disorder were hyperparathyroidism, solid malignancies, and multiple myeloma. The clinical manifestations involved multiple systems, and the complex clinical manifestations easily lead to misdiagnosis. Therefore, the early recognition, emergency treatment, and etiological treatment of hypercalcemic crisis are critical. Comprehensive emergency treatment for hypercalcemic crisis, including fluid hydration, calcitonin, bisphosphonates, and hemodialysis, can rapidly reduce SCa levels and achieve symptomatic relief.

## Figures and Tables

**Table 1:** Distribution of clinical findings and biochemical tests of 155 hypercalcemic crisis patients.

Etiology	Number of cases	The proportion of manifestation (%)					Laboratory evaluation					
		Digestive system	Skeletal system	Nervous system	Urinary system	Cardiovascular system	SCa (mg/dl)	Corrected SCa (mg/dl)	Serum phosphorus (mmol/L)	25-OH vit D3 (ng/ml)	ALP (U/L) (P25, P75)	PTH (pg/ml) (P25, P75)
Hyperparathyroidism	65	82.54	75.38	67.69	63.07	21.54						
Primary	47	76.59	78.72	61.70	68.08	23.40	15.12 ± 2.00	15.40 ± 1.64	0.81 ± 0.19	10.96 ± 6.52	132.40 (102.70, 339.80)	721.70 (318.45, 1351.50)
Tertiary	18	88.88	66.67	83.33	50.00	16.67	14.72 ± 2.04	14.44 ± 2.44	1.00 ± 0.67	12.58 ± 6.33	104.55 (79.85, 162.28)	434.35 (262.32, 833.37)
Solid malignancy	64	71.87	32.81	62.50	50.20	46.87						
Secondary hypercalcemic crisis due to long-term primary tumour history	48	77.08	33.33	66.67	45.83	45.83	15.36 ± 1.92	15.92 ± 2.04	1.12 ± 0.43	14.72 ± 4.09	134.50 (98.40, 224.97)	7.15 (6.21, 9.36)
Hypercalcemic crisis as the first symptom of solid malignancy	16	56.25	31.25	50.00	62.50	50.00	15.84 ± 2.00	16.08 ± 2.04	1.15 ± 0.33	31.81 ± 35.13	115.10 (93.30, 188.90)	8.26- (6.55, 14.56)
Multiple myeloma	14	71.42	78.57	42.86	71.42	35.71	14.84 ± 2.04	15.16 ± 1.96	1.52 ± 0.32	17.82 ± 7.36	92.00 (77.70, 123.70)	14.66 (11.34, 69.77)
Other causes	12	100	33.33	25	33.33	16.67	16.04 ± 1.88	16.48 ± 2.16	1.06 ± 0.34	32.02 ± 39.28	126.90 (68.19, 185.00)	35.71 (20.10, 50.11)
Normal range							8.36–10.16	8.36–10.16	0.89–1.6	20–32	0–130	15–65

The concentration of ALP and PTH was quantitative data with nonnormal distribution, which presented as median (*r*) or interquartile range (P25 and P75).

**Table 2:** Treatment plan and outcome of 155 hypercalcemic crisis patients.

Etiology	Number of cases	The proportion of treatment (%)										Corrected SCa (mg/dl)		PTH (pg/ml)		Improvement rate (%)	
		Operation	Hydration	Hydragogue	Calcitonin	Bisphosphonate	Glucocorticoids	Hemodialysis	Radiofrequency ablation	Radiotherapy	Chemotherapy	Prior treatment	Post treatment	Prior treatment	Posttreatment	Operation	Others
Hyperparathyroidism	65	56.92	92.31	35.38	35.38	70.77	18.46	4.60	3.07	0	0					100	7.14
Primary	47	65.95	51.06	38.29	40.42	76.59	17.02	6.38	4.25	0	0	15.40 ± 1.64	11.16 ± 1.36	721.70 (318.45, 1351.50)	15.22 (10.23, 37.28)	100	12.50
Tertiary	18	33.33	55.55	27.77	22.22	55.55	22.22	0	0	0	0	14.44 ± 2.44	10.44 ± 0.88	434.35 (262.32, 833.37)	15.81 (13.1, 103.41)	66.67	0
Solid malignancy	64	2	68.75	78.12	50.00	75.00	50.00	6.20	0	4.60	50.00					100	67.74
Secondary hypercalcemic crisis due to long-term primary tumour history	48	2	62.50	77.08	41.60	75.50	50.00	2.00	0	6.25	62.50	15.92 ± 2.04	11.08 ± 2.56	7.15 (6.21, 9.36)	81.27 (73.45, 89.09)	100	58.69
Hypercalcemic crisis as the first symptom of solid malignancy	16	0	87.50	81.25	75.00	75.00	50.00	18.75	0	0	12.50	16.08 ± 2.04	9.92 ± 1.96	8.26 (6.55, 14.56)	7.26 (3.21, 9.10)	0	87.50
Multiple myeloma	14	0	50.00	64.28	57.14	71.43	71.43	28.57	0	0	71.43	15.16 ± 1.96	9.56 ± 1.44	14.66 (11.34, 69.77)	14.70 (11.27, 30.10)	0	78.57
Normal range												8.36–10.16	8.36–10.16	15–65	15–65		

## Data Availability

All data generated or analysed during this study are included within the article.

## References

[1] Medina Ortega L., Feijoo Massó C., Tolosa Vilella C. (2018). Parathyroid crisis: hypercalcemic as a medical emergency. *Medicina Clínica*.

[2] Omotosho Y. B., Zahra F. (2023). *Resistant Hypercalcemic*.

[3] Jiajue R., Song A., Wang O., Li W. (2020). Persistent hypercalcemic crisis and recurrent acute pancreatitis due to multiple ectopic parathyroid carcinomas: case report and literature review of mediastinal parathyroid carcinoma. *Frontiers in Endocrinology*.

[4] Villalba-Ferrer F., Valderas-Cortés G., Basés-Valenzuela C., Río G. A. G. D., Villalba-Segarra A., Zaragoza Fernández C. (2020). Hypercalcemic crisis due to primary hyperparathyroidism resistant to medical treatment. *Cirugia y Cirujanos*.

[5] Turan U., Kilavuz H., Irkorucu O. (2021). Clinical features of hypercalcemic crisis in primary hyperparathyroidism. *Acta Endocrinologica*.

[6] Walker M. D., Hypercalcemia E. S., Review A. (2022). *The Journal of the American Medical Association*.

[7] Zhang J. Z., Ke L., Li G. (2017). Clinical Characteristics of hyperparathyroidism complicated with pancreatitis:a single institution experience with 5 Patients. *Chinese Journal of Pancreatology*.

[8] Jacobi J. (2019). Management of endocrine emergencies in the ICU. *Journal of Pharmacy Practice*.

[9] Xia X., Cao J., Long C., Chen X., Zheng J. (2023). Clinical characteristics of hypercalcemic crises in a tertiary children’s hospital. *Endocrine*.

[10] Bilezikian J. P., Khan A. A., Silverberg S. J. (2022). Evaluation and management of primary hyperparathyroidism:summary statement and guidelines from the fifth international workshop. *Journal of Bone and Mineral Research*.

[11] Xu Q. Q., Kong N., Liang C. R. (2021). Value of ultrasonography, radionuclide imaging and CT in preoperative location diagnosis of primary hyperparathyroidism. *Chinese Journal of General Surgery*.

[12] Cowan A. J., Green D. J., Kwok M. (2022). Diagnosis and management of multiple myeloma:A review. *The Journal of the American Medical Association*.

[13] Bentata Y., El Maghraoui H., Benabdelhak M., Haddiya I. (2018). Management of hypercalcaemic crisis in adults: current role of renal replacement therapy. *The American Journal of Emergency Medicine*.

[14] Brown J. P., Reid I. R., Wagman R. B. (2014). Effects of up to 5 years of denosumab treatment on bone histology and histomorphom-etry:the FREEDOM study extension. *Journal of Bone and Mineral Research*.

[15] Chen Y., Ling X., Kong W., Wang S., Yu Z. (2020). Pazopanib as salvage therapy in metastatic renal cell carcinoma with hypercalcemic crisis and renal insufficiency: a case report and literature review. *Translational Cancer Research*.

[16] Wei L. F., Li R. S., Zhao H. (2021). Long-term clinical evaluation on total parathyroidectomy in patients with secondary hyperparathyroidism. *Chinese Journal of General Surgery*.

